# Aboriginal Community Controlled Organisations Leading the Way in Child Health Research

**DOI:** 10.1007/s10900-024-01433-7

**Published:** 2025-01-20

**Authors:** Anita Pickard, Thomas Stubbs, Emily Carter, Lauren Rice, Sue Thomas, Jadnah Davies, June Oscar, Alexandra Martiniuk, Elizabeth J. Elliott

**Affiliations:** 1 Faculty of Medicine and Health, School of Public Health, The University of Sydney, Sydney, NSW Australia; 2 Faculty of Medicine and Health, Specialty of Child and Adolescent Health, The University of Sydney, Sydney, NSW Australia; 3 Marninwarntikura Women’s Resource Centre, Marulu Team, Fitzroy Crossing, WA Australia; 4 Kid’s Research, Sydney Children’s Hospitals Network, Westmead, NSW Australia; 5 Dalla Lana School of Public Health, The University of Toronto, Toronto, ON Canada

**Keywords:** Aboriginal and Torres Strait Islander Peoples, Indigenous, Fetal alcohol spectrum disorders, Child health, Adverse childhood experiences, Health services

## Abstract

Historically, Aboriginal and Torres Strait Islander research in Australia has adhered to Western research paradigms and contributed to the adverse impacts of colonisation. However, recent developments driven by Aboriginal and Torres Strait Islander people and scholars, and development of ethical guidelines for research, have promoted a more inclusive and collaborative research landscape. In this study, published papers and internal documents arising from a long-term partnership between Marninwarntikura Women’s Resource Centre (MWRC) and the University of Sydney (USYD) from 2009 to 2023 were analysed using the Aboriginal and Torres Strait Islander Quality Appraisal Tool and consultations with project partners. Every project had Aboriginal and Torres Strait Islander governance, leadership, and inclusive consultation; was driven by community-identified priorities, built local capacity, and benefitted the community. There was an increase in the use of Indigenous research paradigms and data/intellectual property (IP) agreements over time, reflecting responsiveness to community leadership. This partnership exemplifies community-driven, co-designed, Aboriginal-led health research and serves as a model for effective collaboration with Aboriginal and Torres Strait Islander communities.

**Supplementary Information:**

The online version contains supplementary material available at 10.1007/s10900-024-01433-7.

## Introduction

Aboriginal and Torres Strait Islander peoples^1^ have shown resilience in the face of historical and ongoing adversity and discrimination. Enduring connection to culture, community, and land has contributed to their individual and collective strength and healing [1]. However, they also experience the enduring impacts of colonisation through trauma and systemic racism, which contribute to health inequalities and are exacerbated by reduced access to high-quality and culturally appropriate health services [2–5].

Research has inadvertently reinforced historical injustices against Aboriginal and Torres Strait Islander people. Colonial settlers used research to justify seizing Indigenous lands and the exploitation and oppression of Indigenous people [6]. Additionally, research was used to further colonial interests by perpetuating false, negative stereotypes about Indigenous people, denigrating Indigenous cultures, and fuelling racism and discrimination among non-Indigenous populations [7–9]. In Australia, colonisation and ‘Western’ research has left Aboriginal and Torres Strait Islander people with a profound mistrust and suspicion of non-Indigenous research and researchers [10].

Although research practices have been reforming since colonisation, researchers still face challenges to rectify past injustices and improve current practices [11]. For instance, Aboriginal and Torres Strait Islander people have historically been the ‘subjects’ in research conducted by Western investigators; research objectives, methods, and interpretations have originated from Western scientific traditions. As such, research written in Standard Australian English about Aboriginal and Torres Strait Islander identities, cultures, and stories has been dominated by non-Indigenous ‘Western’ worldviews, ultimately displacing Aboriginal and Torres Strait Islander voices [12]. It has also been questioned as to whether the abundant research on Indigenous peoples has addressed Indigenous priorities, or has been translated into policies or practices that contribute to meaningful improvements in health and wellbeing for communities [13–16]. The Lowitja Institute, Australia’s national Aboriginal and Torres Strait Islander health research organisation, has driven change by demanding that researchers consider knowledge translation and the potential for maximising benefits to Aboriginal and Torres Strait Islander health during research planning and funding phases [13, 14, 17].

Recognition of these issues led to efforts to reform research practices and uphold the rights and preferences of Aboriginal and Torres Strait Islander people. In 1999, the Australian Institute for Aboriginal and Torres Strait Islander Studies (AIATSIS) published ethical guidelines for Aboriginal and Torres Strait Islander research, repositioning Aboriginal and Torres Strait Islander people from subjects to partners in the research process [18, 19]. The National Health and Medical Research Council’s (NHMRC) *Values and Ethics: Guidelines for Ethical Conduct in Aboriginal and Torres Strait Islander Health Research,* published in 2003, also played a role in formalising standards and emphasising the importance of community engagement, informed consent, cultural respect, and benefits for Aboriginal and Torres Strait Islander communities [20]. However, a gap persisted between guideline recommendations and research governance, accountability, and practices, with new studies failing to implement real changes apart from adhering to guidelines for ethical approval [11, 21]. Indeed, a recent review of health research conducted in the Kimberley, Western Australia, between 2006 and 2020 found that most studies were not initiated in the region, did not involve communities or demonstrate benefits, and lacked accountability [22].

Alongside the development of formal guidelines, the work of Aboriginal and Torres Strait Islander scholars and researchers, such as Professor Pat Dudgeon and Professor Sandra Eades, have shaped Indigenous health research discourses, narratives, and methodologies [23]. Indigenous research methods, grounded in Indigenous standpoint theory and Indigenous knowledge systems, emphasise self-determination and Indigenous leadership in the initiation, implementation, translation, and sovereignty of research [24]. This approach requires true collaboration throughout the research process, with Indigenous leadership ensuring research practices aligns with identified priorities for, and ways of knowing, in partner communities. Challenging dominant ‘Western’ paradigms, promoting participatory approaches and Indigenous research methods, and bringing a renewed focus to collaborative, relationship-building processes has improved the quality of Indigenous health research and contributed to de-colonisation and healing [6, 7, 21, 24–26]. Additionally, Aboriginal and Torres Strait Islander researchers such as Professor Peter Yu and Professor Ray Lovett have called for greater Indigenous control over data collection, access, analysis, and storage to allow for genuine self-determination and alignment of research with Indigenous priorities [27, 28].

Updated guidelines such as the AIATSIS Code of Ethics for Aboriginal and Torres Strait Islander Research (2020) [18] and the 2018 NHMRC guidelines for research with Aboriginal and Torres Strait Islander people [29] reflect developments in these critical areas of research including promoting Aboriginal and Torres Strait Islander self-determination, leadership, data ownership, governance, the use of inclusive consent processes and Indigenous methodologies, and research that benefits that are valued by communities. Indigenous and decolonising approaches in research also lead to capacity building in Indigenous community members and researchers who are embedded in research and provided with employment and training opportunities [10, 30].

A recent example of community-led research is “Yuwaya Ngarra-li”, a partnership between the Dharriwaa Elders Group in Walgett and the University of New South Wales (UNSW) [31]. After a successful research collaboration investigating the criminalisation of Aboriginal people with mental and cognitive disability, the Dharriwaa Elders Group invited the UNSW to collaborate with a shared commitment to systemic change. The partnership conceptualised the model ‘CommUNIty Led Development’ which centres the leadership of local Aboriginal Community Controlled Organisations (ACCO) and draws on the skills and expertise of research institutions to create sustainable change [31].

While the efforts of Aboriginal and Torres Strait Islander leaders are shifting the way Aboriginal and Torres Strait Islander research is conducted in Australia, this is not happening as quickly as it should. Three decades after the first guidelines on ethical Aboriginal and Torres Strait Islander health research were published, a 2021 national survey of researchers working in Aboriginal and Torres Strait Islander health revealed that although the NHMRC guidelines were widely used, Aboriginal and Torres Strait Islander governance and participation were inadequate, and significant challenges remained [22], [32]. To overcome these issues, genuine and effective partnerships are needed that ensure Aboriginal governance and leadership, operationalise these principles and ensure critical reflection on such practices to guide research. The need for genuine partnerships is not unique to research, and priority reform 1 of the 2020 Closing the Gap Targets emphasise the need for government to work in genuine partnerships with Aboriginal and Torres Strait Islander people and communities to ensure shared decision-making and place-based solutions [33].

In this paper, we highlight the history and ongoing collaboration between Marninwarntikura Women’s Resource Centre (MWRC) in the remote Fitzroy Valley region of Western Australia and the University of Sydney (USYD), as another example of an ethical, community-led research partnership.

## Setting

Fitzroy Crossing is located 400 km east of Broome and is the service town for the surrounding Fitzroy Valley, home to approximately 3500 people belonging to five predominant language groups (Bunuba, Walmajarri, Gooniyandi, Nyikina and Wangkatjungka). There are 32 active very remote communities in the Fitzroy Valley and 80% of the population are Aboriginal [34]. The communities of the Fitzroy Valley have shown great resilience and continue to maintain traditional cultural practices in the face of the historic and ongoing impacts of colonisation. In 2007, alcohol related harms were widespread and the community was in crisis [35–38]. Senior women in the Fitzroy Valley gathered at a Women’s Bush Camp to address alcohol and its impact in the community. In 2007 they successfully lobbied for restrictions on the sale of take-away liquor containing more than 2.7% alcohol from the two pubs in Fitzroy Crossing. The leadership of Ms June Oscar (AO) and Ms Emily Carter (AM) and the community’s journey to supporting the restrictions was documented in the film Yajilarra and elsewhere [37]. External evaluations by the University of Notre Dame identified significant benefits of the restrictions to the community [38–40].

## The Partnership

Following the implementation of the alcohol restrictions, Aboriginal women in the Fitzroy Valley became increasingly concerned about the relationship between alcohol use in pregnancy and the delayed development they observed in some of their children. In 2008 MWRC, represented by CEO June Oscar (AO), and Nindilingarri Cultural Health Services (NCHS), represented by CEO Maureen Carter, invited the USYD, represented by Professor Elizabeth Elliott (AM) from the Faculty of Medicine’s Discipline of Paediatrics, and Child Health and Professor Jane Latimer (AO) from the George Institute for Global Health, to the Fitzroy Valley. They formed a partnership to implement the Marulu Strategy [38] to address fetal alcohol spectrum disorders (FASD) and early life trauma^2^ (ELT) in children. Marulu is a word in the Bunuba language meaning ‘precious, worth nurturing’, which the community use to refer to their children. The strategy was a collaborative initiative involving community leaders, organisations, service providers, government, and research institutions. These groups work collectively to develop effective approaches to address FASD and ELT, spanning prevention, diagnosis, community education and capacity building, service improvements, and therapeutic support for families. ACCOs in the Fitzroy Valley are now recognised internationally for their early initiatives to reduce alcohol-related harms and address FASD and ELT [35]. The partnership between MWRC, now led by CEO Ms Emily Carter, and the USYD has since conducted multiple research projects to build evidence and improve the health and wellbeing of children, adolescents, and families. This paper presents a summary of these projects, highlights their research impacts, and critically appraises their adherence to Aboriginal and Torres Strait Islander research guidelines and principles.

## Methods

Two authors (AP & TS) not previously involved in the partnership’s research reviewed published papers from the projects led by MWRC or MWRC-NCHS and internal documents such as grant proposals, memorandums of understanding (MOUs), and published reports identified by project partners. They also conducted a consultation process with key stakeholders from both organisations to obtain their reflections on the impacts of the partnership. To document and evaluate how the research partnership aligns with Indigenous preferences and perspectives, the Aboriginal and Torres Strait Islander quality appraisal tool [41] was used to assesses the quality of projects against 14 questions covering: Indigenous leadership and governance, community engagement and consultation, implementation of research and respect of cultural protocols, Indigenous research paradigms, intellectual and cultural property rights, translation of findings in policy and practice, capacity building and benefits to participants and communities involved [41, 42]. Both reviewers applied the quality appraisal tool and any discrepancies in results were discussed and resolved.

## Results

**Fig. 1:**
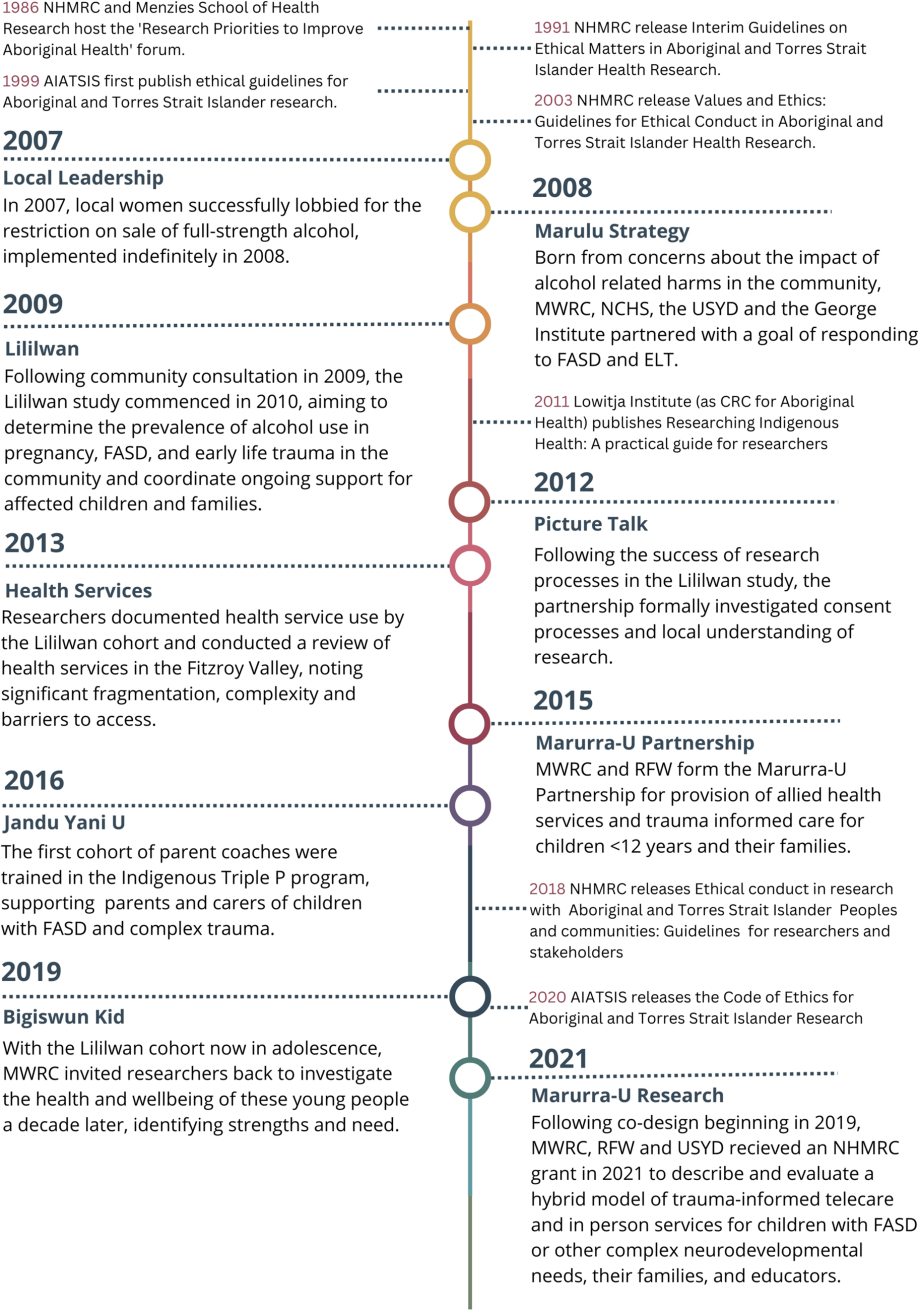
Timeline of research projects led by Aboriginal Community Controlled Organisations in the Fitzroy Valley, Western Australia. Figure was made using Canva

Since 2009, MWRC has collaborated with the USYD on six research projects that embed ethical research practices and Aboriginal ways of working. Research is led by the community and focused on the needs and priorities identified by local leaders, with emphasis on direct and immediate benefits to the study participants and wider community. Each project’s aim and funding sources are shown in Table 1. A summary of each project in chronological order, followed by a review of short- and long-term impacts of each project, is documented in Table 2. Results of the analysis using the Aboriginal and Torres Strait Islander quality appraisal tool are presented in Table 3. A timeline of projects and relevant ethical or research guidelines is shown in Fig. 1.

**Table 1:** Research projects led by MWRC or MWRC and NCHS* and their aims and funding sources

Project name	Aims and funding source
Lililwan (all the little ones)*	To determine the prevalence of alcohol use in pregnancy, FASD and ELT in children aged 7–9 years, and document the impacts of prenatal alcohol exposure (PAE) on child development and learning. (NHMRC: #1,024,474; DOHA; FaHCSIA)
Picture Talk*	To learn what research means to Aboriginal people of the Fitzroy Valley, who holds and shares knowledge and how new knowledge is generated and passed on; to explore experiences of, and attitudes towards, research, the process of community engagement and consultation, and the preferred process of obtaining community and individual consent for research. (NHMRC: #1,024,474; DOHA; FaHCSIA)
Health Services 2013	To document past health service use and hospitalisation of children in the Lililwan cohort; to identify and map child health services in the Fitzroy Valley following the Lililwan project; and to identify service limitations and barriers to service access and delivery. (NHMRC: #1,024,474; DOHA; FaHCSIA)
Jandu Yani U (for all families)	To train and accredit community members to implement a locally adapted version of the Indigenous positive parenting program (Triple P) in the Fitzroy Valley; to support caregivers and families, particularly those with children with complex needs; and to evaluate the program’s effectiveness in remote Aboriginal communities. (NHMRC: #1,068,620)
Bigiswun Kid (adolescent)	To identify the needs of adolescents and build knowledge to inform services to improve the health and wellbeing of adolescents in remote Aboriginal communities. (Australian Rotary Health, Ian Potter Foundation: #31,110,414, Healthway: #33,726), Lowitja Institute, USYD, Westpac—LR). Research outcomes of this project led to projects to develop FASD resources for Health Professionals (Funded by NDIA) and examine barriers and enablers to accessing National Disability Insurance Scheme (Funding NDIA)
Marurra-U (to embrace with love and care)	To develop, implement and evaluate a model of wrap-around specialist paediatric and allied health care in the Fitzroy Valley in collaboration with Royal Far West and incorporating trauma-informed, culturally appropriate services delivered in communities in person and/or virtually e.g. telecare. (NHMRC: #1,171,880)

**Table 2:** Examples of Impacts from community-led research projects

Project	Short-term impacts	Long-term impacts
Lililwan	Children received comprehensive, multidisciplinary neurodevelopmental and health assessments (paediatric, ear, eye, psychosocial and cognitive, physiotherapy, occupational therapy, speech pathology, mental health). FASD diagnoses and > 400 referrals to allied health, paediatric, child psychology or psychiatric services for ongoing care and supports were given [63] Immediate treatment for children with acute illness e.g. otitis media, skin sores, respiratory infections [45] Education and health management plans for each child shared with parents, schools and health services (with parental consent) [45] Follow up support from internationally recognised FASD educator for children with FASD diagnoses and their families [63] Training and development for 'community navigators’, local health professionals and teachers at participating schools [63] MWRC and NCHS developed resources to raise community awareness including the films Marulu [98] and Tristan [76, 99]. Tristan was shown at the United Nations Permanent Forum for Indigenous Issues, New York 2012 where Aboriginal researchers presented the study results [99] Training of local Aboriginal people, health professionals, teachers, community workers to build research and clinical capacity [45, 66]	MWRC used findings to obtain funding to establish three new services: 1. The Marulu Team to support the Lililwan cohort and other children with complex needs in the Fitzroy Valley; 2. The Baya Gawiy Child and Parent Centre, which houses multiple in-house and outreach services, and; 3. The Early Childhood Learning Unit (within Baya Gawiy) [100] NCHS submission to the House of Representatives Inquiry into the high level of involvement of Indigenous juveniles and young adults in the criminal justice system, 2009 [101] Presentation of study data to the House of Representatives Inquiry into the prevention, diagnosis and management of FASD at a bush meeting, Mimbi Caves [102]. Data was also presented in person, submitted, and included in the report on *‘FASD, the Hidden Harm’*, 2012 [103]. Following this inquiry, the Australian government announced $9.2million in funding over fours years under the FASD Action Plan [104] Inclusion of data in the WA legislative assembly Inquiry into Improving Educational Outcomes for Western Australians of All Ages in the *Invisible Disability Report*, 2012 [105] Submission to the Legislative Assembly of the Northern Territory Select Committee on Action to Prevent Foetal Alcohol Spectrum Disorder, 2014 [106] Submission to The House of Representatives Inquiry into the harmful use of alcohol in Aboriginal and Torres Strait Islander communities, 2014 [107] Submission to the Senate Finance and Public Administration References Committee’s Inquiry into Legal Assistance Services, 2015 [108] MWRC presentations to coronial inquests relating to alcohol use, FASD and intergenerational trauma in the Kimberley 2008, 2017 [109, 110] Informed the Australian guidelines to reduce health risks from drinking alcohol, 2020 [111] Informed the Australian guide to the diagnosis of FASD [73]. Data used to advocate for motor assessment to be a standard component of FASD diagnostic assessment [52, 112] NCHS used the data to inform health promotion materials on PAE harms and coordinate activities on International FASD Awareness Day. This has become an annual event in Fitzroy Crossing [113] Data informed the National FASD Strategic Action Plan 2018–28 [74] Data informed advocacy for pregnancy warning labels on all alcoholic beverage containers and packages, introduced under the Australia and New Zealand Food Standards Code from 31 July 2023 [114] MWRC developed three resources for teachers and early childhood educators on FASD and ELT, 2014, 2018, 2023 [77–79, 115], and delivered ongoing professional development to Government, Catholic and Independent schools in the Kimberley and beyond (Personal communication, ST, 2023) MWRC, NCHS, the USYD and the George Institute developed the film ‘The story of alcohol use in pregnancy’ to educate health professionals and the broader Australian community [80] Study data are repeatedly drawn upon to support continuation of restrictions on take-away alcohol when these are challenged and enforcement of laws to enable ‘dry’ households and ‘dry’ communities [36, 116, 117] EC and ST are members of the group that provided expert advice and informed development of Strong Born resources by the National Aboriginal Community Controlled Health Organisation as part of the Foundation for Alcohol Research and Education *‘Every Moment Matters’* national awareness campaign on FASD and alcohol harms in pregnancy, 2022 [118] Funding was provided by the Kimberley Brain and Mind Foundation for educational resources and opportunities for training to enable police to understand and respond to people with FASD Funding from the Kimberley Brain and Mind Foundation was provided to support a program of riding for the disabled in Fitzroy Crossing Development of educational resources on FASD and delivery of educational programs for other Aboriginal communities including Cherbourg, Queensland [119] The Lililwan project has been referenced 30 times in Hansard across records from Joint Committee, the House of Representatives and the Senate, highlighting the significant influence this project and ongoing work has had in the Australian Parliament and policy [120] The Marulu strategy remains a leading example of community-led intervention and has been described in the book *Learning from 50 Years of Aboriginal Alcohol Programs* [36]: “The Marulu Strategy is ongoing; it remains the most comprehensive, community-led intervention anywhere in Australia for preventing and managing FASD and providing support to families with FASD-affected children”. The Marulu strategy is regarded as best practice in tackling FASD [71]
Picture Talks	Increased local research capacity through employment of community navigators [82] Provided a voice to local communities on research practices, including on recent projects and processes [82] Participants could raise issues they felt were most pressing to the community and advocate for supports they needed [67] The Picture Talk project strengthened relationships between the researchers and communities in the Fitzroy Valley and provided practical advice on respectful community engagement	Advocacy for changes to existing guidelines for research with Indigenous people [67] Practical guidance for ongoing Indigenous research in partnerships between communities and external partners. [67]
Health Services	Serves as the only existing outline of health service use, hospitalisation, and available child health services in the Fitzroy Valley [86–89] Scoping review identifies challenges to remote services provision [89] Informed service planning for MWRC (Personal Communication ST, August 2023)	Identified health service needs and challenges in the Fitzroy Valley, informing service planning [86] Informed the three-year formal review of the National Fetal Alcohol Spectrum Disorder (FASD) Action Plan 2018 – 2028 [121] Systematic review provided recommendations for addressing barriers to health service provision and access to health services in remote Indigenous communities [89]
Jandu Yani U	Co-designed a model of community engagement for research [94] Training and accreditation by Triple P International of 38 (24 Aboriginal) parent coaches from local organisations in the Fitzroy Valley in the use of a locally adapted version of Indigenous Triple P (many had limited prior educational opportunities). Reported increase in empowerment and skills Parent coaches delivered the Triple P program to 30 families and over 530 adults were informally engaged [91, 92] Increased parent/carer empowerment, skills, knowledge and confidence; improved paternal social and emotional wellbeing; enabled more effective parenting practices; and improved child behaviours [91, 92] Networking between service providers working in the Fitzroy Valley during Triple P Parent coach training [91]	Ongoing support was provided by an experienced Triple P practitioner residing in community for an extended period, supporting the parent coaches [91] Learnings from the parenting coaching continue to inform engagement with families through MWRC’s other services and parent coaches implement informal training with families (Personal Communication with ST, 2023) and will inform delivery of parent supports in other Aboriginal communities Programming for families throughout Fitzroy Valley was maintained, supported by funding from the Marulu Strategy [94] A partnership agreement template was developed for MWRC to support positive ways of working in future endeavours [94] Submission to the Senate Inquiry into Effective Approaches to Prevention, Diagnosis and Support for Fetal Alcohol Spectrum Disorder, 2021 [71]
Bigiswun Kid	Adolescents were assisted to obtain identification documents such as birth certificates, Medicare cards, bank accounts, tax file numbers. These were used to help young people obtain a driver’s license Adolescents were provided with practical assistance in applying for jobs (resume writing, interview practice, accompanying to agency) Young women attended weekly, on-country art therapy groups Young men and women were assisted in accessing existing mental health, antenatal, and other health services The team partnered with community members to run a range of on-country activities and camps, including a whole of community wellbeing camp Community members received locally adapted training at suicide prevention workshops Young people were assisted in applying for housing; advocacy to policymakers regarding housing needs Provided infant and child development workshops in 5 communities People with disability and their families were supported to navigate the NDIS application process and access NDIS-funded supports [96] The Fitzroy Valley was chosen as one of two regions highlighted in public hearing 25 of the Royal Commission into the Violence, Abuse and Neglect of People with Disability, which focused on the operation of the NDIS for Indigenous people with disability in remote and very remote communities. People with disability were supported by MWRC and USYD staff to participate in this hearing and provide their evidence [122]	Preliminary Bigiswun Kid project findings were used to help MWRC secure a National Indigenous Australians Agency grant to design a supported work program. (Personal Communication with LR, 2023) Advocacy to policymakers resulted in MWRC securing funding from the WA Mental Health Commission to design and pilot a youth social and emotional wellbeing service Worked with the Department of Transport to adapt their policies and procedures to help support young people in the Fitzroy Valley obtain a driver’s licence The MWRC-USYD partnership secured an NDIS Information, Linkages and Capacity Building grant to increase mainstream health services for people with disability MWRC staff are developing resources for health professionals and police to work with youth with FASD, building on the research findings and understandings from the lived experiences of community members (Personal communication, ST, 2023)
		Bigiswun findings led to the NDIS consultation project. The Project report *People Don’t Know What Good Looks Like* on disability services in the Fitzroy Valley, 2021 [96]*,* was shared widely and presented to the national FASD Advisory Group and the Federal Minister for the NDIS; led to growing community and organisational knowledge about the NDIS; and provided a platform to share understandings from lived experience and advocate to the NDIA for better NDIS access from an informed position Presentation to the Royal Disability Commission and Joint Standing Committee on access to NDIS resulted in an invitation to an Advisory Group to inform drafting of NDIS policies for remote communities [122] Increased community member and local ACCO’s understanding of the NDIS Information captured allowed the NDIA to understand the needs of the community and develop a place-based design for the Fitzroy Valley The NDIA adapted a range of processes to help address the barriers to obtaining NDIS support faced by people in very remote regions like the Fitzroy Valley The report findings continue to be used to raise the voices of people with disability in remote Aboriginal communities Additionally, funding from the NDIA was awarded to further investigate FASD resources for health professionals [97] Interviews with health professionals and community members around disability and FASD aimed to improve understanding between western health services and Aboriginal communities accessing such services. These will inform development of locally relevant resources for health professionals and communities
Marurra-U	Children in the Fitzroy Valley receive occupational therapy and speech pathology support via telecare (Personal communication, EC, ST, and RFW Sept 2023) Teachers and staff at schools and organisations in the Fitzroy Valley receive trauma-informed specialist training and professional support in working with children with FASD, ELT and complex social-emotional needs Audit and adjustment of spaces such as the Women’s Shelter, Department of Communities meeting spaces, schools, playgrounds, and MWRC areas with a trauma-informed lens Support for the Marulu team at MWRC with trauma-informed learning, coaching, evidence-informed practices and supervision Children and families receive intensive trauma-informed care with allied health professionals at in-person camps. Parents and carers reported an increased understanding of child brain development and behaviour, and strategies to support their children’s wellbeing and development. Parents and carers also felt their children benefitted from attending camp with gains made to social emotional and wellbeing positive interactions. (Personal communication JD, April 2024) Fitzroy Valley District High School received review of teacher self-efficacy for working with children with complex needs and are supported to implement school-wide measures to support children and families through teacher coaching and engagement with the leadership team	Capacity building of local health professionals and educators with trauma informed training (Personal Communication, EC, ST and RFW 2023) Knowledge sharing and learning through community member visits to RFW premises in Sydney Practical advice and support to incorporate new and emerging knowledge across different MWRC teams, including the Strengthening Families team and Baya Gawiy early childhood teams Supported a post-flood needs assessment by UNICEF, advocating for supports for, and highlighting the needs of, children, families and services [123] Interviews with team members document the partnership between a western allied health service and community-controlled organisation to facilitate health services, despite challenges with funding, natural disasters, COVID-19 and other barriers. A report on parent and family experiences at the Marurra-U Family Camp and interviews with teachers capture the experiences of participants engaging with the Marurra-U team

**Table 3:** Aboriginal and torres strait islander quality appraisal tool assessment

Question	Lililwan	Picture Talk	Health Services	Jandu Yani U	Bigiswun	Marurra-U
Did the research respond to a need or priority determined by the community?	Yes	Yes	Yes	Yes	Yes	Yes
Was community consultation and engagement appropriately inclusive?	Yes	Yes	Yes	Yes	Yes	Yes
Did the research have Aboriginal and Torres Strait Islander research leadership?	Yes	Yes	Yes	Yes	Yes	Yes
Did the research have Aboriginal and Torres Strait Islander governance?	Yes	Yes	Yes	Yes	Yes	Yes
Were local community protocols respected and followed?	Yes	Yes	Yes	Yes	Yes	Yes
Did the researchers negotiate agreements regarding rights of access to Aboriginal and Torres Strait Islander’s existing intellectual and cultural property?	Partial	Partial	Partial	Partial	Yes	Yes
Did the researchers negotiate agreements regarding rights of access to Aboriginal and Torres Strait Islander peoples’ ownership of intellectual and cultural property created through the research?	Partial	Partial	Partial	Partial	Yes	Yes
Did Aboriginal and Torres Strait Islander peoples and communities have control over the collection and management of research materials?	Yes	Yes	Yes	Yes	Yes	Yes
Was the research guided by an Indigenous research paradigm?	Partial	Yes	Partial	Yes	Yes	Yes
Does the research take a strengths-based approach, acknowledging and moving beyond practices that have harmed Aboriginal and Torres Strait Islander people in the past?	Partial	Yes	Partial	Yes	Yes	Yes
Did the researchers plan and translate the findings into sustainable changes in policy and/or practice?	Yes	Yes	Yes	Yes	Yes	NA
Did the research benefit the participants and Aboriginal and Torres Strait Islander communities?	Yes	Yes	Yes	Yes	Yes	Yes
Did the research demonstrate capacity strengthening for Aboriginal and Torres Strait Islander communities?	Yes	Yes	Yes	Yes	Yes	Yes
Did everyone involved in the research have opportunities to learn from each other?	Yes	Yes	Yes	Yes	Yes	Yes

*NB Further explanation of the QAT appraisal can be found in supplementary file 2

### Lililwan Project

Under the Marulu strategy, the MWRC, NCHS, USYD and George Institute research partnership conducted the Lililwan project to determine the prevalence of alcohol use in pregnancy, FASD, ELT, and other neurodevelopmental challenges in children born in 2002–2003 (Table 1) [43, 44]. Of the children who were living in the Fitzroy Valley at age 7–9 years, 55% had experienced PAE, nearly one-fifth (19%) had FASD, and ELT was almost universal [45–48]. Although strengths included gross motor skills, many children had documented fine motor impairment, challenging behaviours, and learning and developmental delays [49–62]. During this ‘research in action’, all children who participated in the Lililwan project were provided comprehensive health assessments, treated for acute and chronic medical problems, and provided with referrals and individualised health and education management plans [63–65] (Table 2). MWRC used the project findings to obtain funding to establish the Marulu Team to work with families on strategies to coordinate ongoing education and support for the Lililwan cohort and other children and families with ELT and complex needs [66].

The Lililwan project embedded the principles of an Indigenous and decolonising approach, guided by the NHMRC 2003 and AIATSIS 1999 guidelines [19]. The project was initiated and led by the Aboriginal community; all research materials were co-designed with community members; and all contact between researchers and participants was facilitated by ‘community navigators’- local, respected community members with knowledge of language and cultural protocols [63]. Data is owned by the community; all research presentations, films, reports and publications received community approval before release; and all grants, ethics applications and publications named Aboriginal researchers. The project was described by Australia's Aboriginal and Torres Strait Islander Social Justice Commissioner at the time, Mick Gooda, as “a genuine partnership—where research is done with the community and not just about the community; setting an example to the rest of Australia as a process guided by meaningful, respectful engagement and collaboration” [38]. Community members reflecting on the Lililwan project say, “it was not referred to as a visiting project, it was referred to with ownership by the community” [67, 68].

The Lililwan project was the first population-based study of FASD prevalence in Australia using active case ascertainment. It provided accurate data on rates of PAE, FASD and ELT and allowed the research partnership to advocate and plan for health services in the community [69, 70]. Alongside immediate clinical support for children and families, the study prompted the first parliamentary Inquiry into FASD. It informed subsequent Inquiries [71, 72] and the development of the Australian Guide to the Diagnosis of FASD [48, 73] and led to the appointment of EE as Chair of a national FASD advisory committee for the federal government [74]. Additional ongoing impacts from this project [75] include increased community, national, and international awareness of alcohol harms [76–80]; decreased rates of alcohol use in pregnancy locally [81]; capacity building of parents, families, health professionals and educators; and the development of health and education supports (Table 2).

### Picture Talk Project

Following the successful collaboration and community engagement demonstrated in the Lililwan project, Aboriginal leaders at NCHS and MWRC invited USYD researchers to reflect on and investigate the process of community engagement, consultation, and consent for research and to provide practical advice on how to engage with Aboriginal communities in a way that is empowering and culturally respectful [82]. This project was given the name ‘Picture Talk’ to describe the pictorial flip-cards used in the consent process for the Lililwan project [82]. The Picture Talk project identified several crucial factors for informed consent including utilising community navigators for recruitment, offering flexible data collection locations on traditional land, ensuring the presence of a witness during data collection, accommodating flexible timing, demonstrating patience as participants considered project consent, and providing research information in multiple sessions [67, 83, 84]. The importance of investing in building relationships and trust prior to consent gathering and conducting research was also highlighted. Alongside guidelines for research with Indigenous groups, the Picture Talk project provided practical examples and reflections of community members on engagement and consent processes.

Having a project logo designed in the community that visually depicted the study aim assisted with building relationships, trust, and reputation with communities [67, 83, 84]. Other studies led by MWRC, NCHS and the USYD also used logos developed by local artists and names in local languages, aiding in project recognition, trust, and understanding by Fitzroy Valley communities. The Picture Talk project also commissioned local artists to visually represent study findings to enable knowledge sharing with communities [85]. Findings supported advocacy for changes in existing guidelines and directed practice for later projects, including the use of images during community engagement and consent processes and the employment of community navigators [83].

### Health Services Project

In response to the complex, chronic health problems and healthcare needs identified in children in the Lililwan project, Aboriginal leaders initiated the Health Services project to identify and map available child health services in the Fitzroy Valley and their use by the Lililwan cohort [86]. This study revealed that 70% of these children were hospitalised at least once before age seven years, and those children with PAE had higher rates of emergency department presentation [87, 88]. The study highlighted the paucity and variability of services, particularly primary care, for preventable conditions and reliance on emergency services [87, 88]. A scoping review of health services for Aboriginal and Torres Strait Islander children in remote Australia confirmed barriers to effective care including difficulty recruiting health professionals and high turnover; complex and fragmented coordination between government, community-controlled, and health and education sectors; and large distances, remoteness and extreme climate [75]. The Health Services project embedded principles of decolonising research by being requested by community leaders in response to knowledge gained from the Lililwan project. Data from the Health Services project provided the only comprehensive review of the numerous healthcare services in the Fitzroy Valley at the time and informed clinicians and policy makers about the need for improved and better co-ordinated health services.

### Jandu Yani U Project

In response to the Lililwan project findings, the community prioritised the need to assist parents and carers to support children with FASD and complex needs expressed as challenging behaviours. MWRC selected the Indigenous version of the Positive Parenting Program (Triple P) to be delivered in small family groups (Level 4), with the addition of Stepping Stones modules, recommended for children with developmental difficulties. Assisted by the USYD and the University of Queensland, MWRC guided researchers and program designers to adapt the program for the local context [90].

Jandu Yani U provided internationally accredited Triple-P training and certification to 38 community members, or ‘parent coaches’, to enable them to support parents to assist their children and reduce challenging behaviours [91]. The program improved parent mental health, empowerment, and self-efficacy; and enabled individual goals for child behaviour change to be reached. It increased pro-social behaviour and decreased behavioural challenges which were maintained at eight months post intervention [91–93]. This project embedded the Model of Engaging with Communities Collaboratively, a process designed for the effective implementation of health programs [94]. Project design, conduct, and implementation followed participatory research methods and included co-design with Aboriginal leaders, parent coaches, families, and community stakeholders [94]. Not only did the tailored Triple P program benefit parents and the local community, it highlighted MWRC’s ability to act as a local hub for parent supports in the region and coordinate and advocate for support services [91]. The success of the program was attributed to the knowledge and leadership of the community, long-term relationship and trust with the USYD researchers, use of an evidence-based parenting program tailored to the cultural context and an Aboriginal co-trainer, and provision of ongoing onsite community support for parent coaches at the end of the project [91, 92]. The principles of positive parenting taught in Jandu Yani U along with emerging knowledge of trauma and the impacts on the developing brain have since been embedded in the ongoing work of the Marulu team and applied in the support of families with lived experience of FASD and engaged with MWRC programs (Personal Communication with ST and JD, August 2023).

### Bigiswun Kid Project

Senior Aboriginal women from MWRC were aware that some of the Lililwan cohort were thriving in adolescence while others were struggling, so they asked the USYD to help understand why. The Bigiswun Kid project followed up the Lililwan cohort, 10 years later, at age 17–19 years [95]. The project aimed to elevate the voices of young people to understand their needs, determine whether the cohort was able to access the health and education services recommended in the Lililwan project, and identify past or present service gaps and facilitators and barriers to service use. Initial data indicated that many adolescents were resilient and hopeful for their future and have positive peer, family, and cultural connections. Many, however, were unable to access supports and services recommended in the Lililwan management plans, reported mental health concerns, and none had National Disability Insurance Scheme (NDIS) funding (LR, personal communication, 2023). The Bigiswun Kid project is an example of Aboriginal Participatory Action Research. As well as providing knowledge on adolescent needs, study participants (parents and young people) were involved in the design and implementation of the research and were recipients of direct and immediate practical supports provided during data collection, including access to birth certification, health services, on-country and cultural activities and education (Personal communication, LR, 2023). Preliminary study results led to a successful tender by MWRC to the Western Australian Mental Health Commission for pilot funding to address the social and emotional wellbeing of youth in the Fitzroy Valley. From preliminary Bigiswun Kid Project Data, MWRC also secured funding from the National Indigenous Australians Agency to work with local services to design supported work placements for young people in the Fitzroy Valley.

Interviews conducted during the Bigiswun Kid Project highlighted the lack of access to NDIS-funded and related services in the community and resulted in the NDIA inviting MWRC to consult with the Fitzroy Valley community, investigate experiences with the NDIS, and identify barriers and enablers to accessing funding and services [96]. Senior Aboriginal women from MWRC worked with a USYD researcher based in the community (LR) to consult with people with disability and their carers and support them through the NDIS process. This project exemplifies how the MWRC and USYD partnership was responsive to community needs and ensured the voices of community members with disability were included in the design of the Fitzroy Valley NDIS rollout.

The Bigiswun Project also identified the need for locally tailored resources for health professionals. A scoping literature review identified a lack of FASD resources for health professionals in Aboriginal communities [97]. Information from the review and from interviews undertaken to explore the Fitzroy Valley community’s attitudes to and knowledge of disability, will inform development of locally relevant resources for health professionals on disability, including FASD.

### Marurra-U Project

The Marurra-U partnership began in 2015 as a collaboration between MWRC and Royal Far West (RFW), an allied paediatric specialist health service focusing on rural and remote children, to fill service gaps, add trauma-informed knowledge and clinical expertise to the Marulu team, and to help shape the Marurra-U model of care.

Research on the Marurra-U project is funded by an NHMRC Partnership grant led by USYD in partnership with MWRC and Royal Far West. It aims to document and evaluate the ability of a flexible model of care, including telecare, to enhance capacity of service providers to support young children (< 12 years) living with complex neurodevelopmental, health and social-emotional needs (including FASD and ELT), and their families. The Marurra-U partnership team has worked with Yiyili Aboriginal community school, Fitzroy Valley District High School, Yakanarra Community School, and Kulkariya Community School to provide telecare and professional development for teachers and healthcare providers with an emphasis on promoting trauma informed approaches (Personal communication EC, ST, and RFW, 2023). Capacity building for teachers and Aboriginal Educators focuses on strategies to engage and support children with complex needs and is responsive to the school and community context. Flexible program delivery by the Marurra-U partnership team in response to changing community needs and priorities was demonstrated during the COVID-19 pandemic and 2023 floods, including consistent telecare in Yiyili School. In April 2024 the Marurra-U team held a therapeutic family-centred camp for parents/carers and children with complex neurodevelopmental or social emotional needs. Yarning circles with parents and carers who attended camp highlighted carer’s increased understanding of child brain development and behaviours, and benefits for children’s social emotional functioning and wellbeing (Personal communication JD, April 2024).

## Discussion

This paper examines over a decade of collaborative work led by MWRC in partnership with the USYD using the Aboriginal and Torres Strait Islander Quality Appraisal Tool (QAT) [41]. Throughout MWRC's research history, its projects and partnerships have been community-initiated, including co-leadership in early projects from NCHS, demonstrated strong community engagement, and provided tangible benefits to local communities. Projects have also built the capacity of local individuals and organizations, aligning with the AIATSIS and NHMRC guidelines and meeting the NHMRC's Indigenous Research Excellence Criteria [20]. The practices articulated by community were directed by the women’s cultural and community authority, resulting in project ways of working that align with guidelines and principles developed subsequently. Analysis illustrates a genuine commitment to community-led research and an effective, collaborative relationship between the USYD and MWRC that has resulted in short- and long-term impacts for participating communities. Significant government funding for establishing new services, prevention programs, targeted research, and strategies to raise community awareness about alcohol harms has been informed and driven by research led by ACCOs in the Fitzroy Valley [71].

All projects performed well when assessed for cultural integrity using the QAT and each project was driven by community-identified priorities. The concept for each research project originated from community leaders, who identified key needs. Researchers were invited to collaborate on these projects, addressing the priorities set by the community leaders such as FASD, ELT, providing consent for research, child and adolescent wellbeing, and health service needs. Conceptualisation and planning of each project involved extensive consultation with community leaders and organisations, with the research team responding to and privileging community voices in all discussions. Community organisations, particularly MWRC, then led the research partnership, developing governance models for engagement of communities, implementation of research activities, and dissemination of results. Consistently, local Aboriginal researchers were involved as chief investigators and authors on ethics applications, grants, presentations, and publications. Under the guidance of MWRC and through employment of community navigators, local cultural protocols were followed, and research activities were conducted in appropriate ways. The employment of Aboriginal co-researchers and community navigators for each project offered two-way learning between Indigenous and non-Indigenous team members. Community navigators assisted with cultural guidance, introductions to and engagement with community members, translation, and consent processes on all projects, whilst learning about Western research methodologies. The research partnership has produced 36 publications so far, involved the work of five PhD students and over 40 community navigators have been involved in research processes.

Each project had an established MoU with local organisations that outlined the roles and responsibilities for each organisation, the risks, outcomes and benefits of the research, respect for the culture and traditions of the community, and the principles of IP and data ownership. However, these agreements only partially fulfilled the quality criteria for legally binding agreements with regard to existing IP rights with participating communities [42]. The most recent projects, Bigiswun Kid and Marurra-U, do have a binding multiple institution agreement that addresses ownership of existing IP. The partnership has demonstrated growth over time, with the transition in project MoUs from joint ownership of research data by the partnership to IP ownership by the community, represented by MWRC, in later projects. Aboriginal people have always had control of the collection and management of research materials, guided by MWRC, and agreement is required before dissemination of research findings, as outlined in project MoUs. Community navigators lead feedback of results to communities.

Several projects (including Picture Talk, Jandu Yani U and Bigiswun Kid) reported use of Indigenous research methodologies such as the Model of Engaging Communities Collaboratively and Participatory Action Research [94, 95]. Other projects did not explicitly report using an Indigenous paradigm but incorporated methods such as yarning and flexibility in interviews and data collection that allow for storytelling, or adaptation of ‘Western’ research resources [90] and practices that privilege Indigenous ways of knowing, being and doing. Additionally, all projects incorporated Indigenous perspectives through input of Aboriginal researchers, advisory groups, and local leaders in research design and implementation. Moreover, the body of work led by MWRC acknowledges the interconnected aspects of health and wellbeing and how these are influenced by colonisation and inter-generational trauma. The use of assessments that are independent of cultural and language backgrounds (e.g. the Universal Non-verbal Intelligence Test); and cultural visual aids in the consent process (Lililwan, Picture Talk, Bigiswun Kid, Marurra-U), adaption of training materials (Jandu Yani U) and translation of interviews or information into local language (all projects) demonstrate equity and respect and ensure the consent process is ongoing and promotes participant self-determination [10, 25].

The Bigiswun Kid project is an example of Aboriginal Participatory Action Research, where participants (adolescents) influenced the project’s design, were involved in all research activities, and received immediate assistance with their greatest needs [22, 84]. The Bigiswun Kid project demonstrates an immediate impact for participants and MWRC obtained funding to establish and pilot an adolescent social and emotional wellbeing program evidenced by the research, which continues to support young people in the Fitzroy Valley after conclusion of the Bigiswun Kid project. Other research projects also demonstrated sustainable changes in policy and practice, along with immediate benefits to communities. For example, research findings influenced national inquiries into FASD, alcohol use, mental health services, and Indigenous incarceration, and development of the Australian guidelines for the diagnosis of FASD, the National FASD Strategy [74], and commissioned work for the NDIA [73, 75, 96]. Impacts include significant government funding for FASD support services, along with targeted research, clinical services, prevention programs, and community awareness [104]. The Lililwan study was also rated as highly impactful in the inaugural Australian Research Council assessment of university research impact [124]. Under the National Partnership Agreement for Indigenous Early Childhood Development, MWRC set up the Baya Gawiy Child and Family Centre, with research influencing the building design and teaching practices. MWRC has facilitated ongoing learning for parents, educators, and health practitioners in trauma-informed care and support of children with FASD, PAE, and ELT. Research data has been used to support continuation of community-initiated alcohol restrictions despite industry challenges [116, 117].

The transformation in approach to research over time reflects the partnership's commitment to incorporating Indigenous perspectives, knowledge systems, and worldviews in their research, aligning with the evolving thinking and teachings from Indigenous scholars during the same period and leadership from local stakeholders.

This paper has some potential limitations. Firstly, it purposely limits the content to a relatively small number of projects initiated and led by ACCOs in the remote Fitzroy Valley of Western Australia, in partnership with clinical academics from the USYD, over a sixteen-year period. Although research results are unlikely to be replicated in similar remote Aboriginal communities elsewhere, the challenges faced in such communities may be similar and the project findings might be applicable. A strength arising from the collective work is that the Fitzroy Valley community has led the way nationally and internationally on social reform, including the introduction of alcohol restrictions, an effective community governance process (Fitzroy Futures Forum, Women’s Bush Camps), and efforts to address and de-stigmatise FASD. A second limitation of this paper is that most authors were closely involved in the planning, conduct, and reporting of one or more projects, raising the potential for bias in the assessment of projects. A strength, however, is that assessment of projects was performed using a standardised approach with a recognised tool by two researchers who were not involved in the conduct or dissemination of any of the listed projects, though have assisted in planning for the upcoming Marurra-U model evaluation. Demonstrating research impact and the value of research to Indigenous populations has been debated in recent years [13], and capturing impacts and knowledge translation over a long period is a strength of this paper.

## Conclusion

Since 2009, MWRC has led child health research in the Fitzroy Valley. Beginning with the Lililwan project, which provided children with comprehensive neurodevelopmental assessments, through to the Marurra-U project, which will develop a model of care for young children with FASD and complex neurodevelopmental needs, the community has secured funding to implement or continue four new services. Parents, carers and teachers have been supported in managing and helping children with FASD and other types of neurodiversity, and communities have learned about FASD and been empowered to act through the Marulu Strategy. During this time, research co-designed and conducted by the community in collaboration with the USYD contributed to national reviews on child health, particularly regarding FASD, the NDIS, ethical practices in Indigenous research, and national policy. It also empowered ACCOs to advocate for systemic change within government services. Each sequential project addressed priorities identified by community leaders and was embedded in ACCOs who, along with the researchers, took the time for inclusive consultation to ensure a wide range of people across the remote communities guided the research. This was possible due to Aboriginal leaders facilitating extensive community involvement beyond the ACCOs, with large teams of community members involved, consulting, and working on the research projects. Such research was made possible by the long-term, genuine partnership between organisations. This allowed partners to develop strong, trusting relationships, with project teams committed to two-way learning over time.

## Supplementary Information

Below is the link to the electronic supplementary material.

Supplementary file1 (XLSX 27 KB)

Supplementary file2 (DOCX 29 KB)

## Footnotes

1 Note that hereafter, the term “Indigenous” will be used in an international context, “Aboriginal and Torres Strait Islander” refers to the First Peoples of Australia and the people of the Fitzroy Valley in the Kimberley region in Western Australia will be described as “Aboriginal” according to their preference.

2 *Adverse childhood experiences (ACEs) and ELT are closely linked. This team conceptualises that ACEs are events that can increase chances of experiencing ELT and use the term ELT as such in this paper.*
