# Supplementary material for: Aboriginal Community Controlled Organisations Leading the Way in Child Health Research
Source: J Community Health. 2025 Jan 20;50(3):527–45. doi: 10.1007/s10900-024-01433-7 (PMC12069493; doi:10.1007/s10900-024-01433-7)
Supplement: Supplementary file 2 — Supplementary file2 (DOCX 29 KB) [file 10900_2024_1433_MOESM2_ESM.docx]

**Aboriginal Community Controlled Organisations Leading the way in Child Health Research**

Journal of Community Health

Anita Pickard^1,2*^(0009-0006-5988-0120)., Thomas Stubbs^1,2^(0000-0001-5676-4374), Emily Carter^3^_,_ Lauren Rice^2, 4^ (0000-0002-2315-7698), Sue Thomas^3^, Jadnah Davies^3^, June Oscar, Alexandra Martiniuk^1,5^(0000-0003-1368-8206), Elizabeth J Elliott^3, 4^ (0000-0002-6501-5487)

^1^ Faculty of Medicine and Health, School of Public Health, The University of Sydney, Sydney, New South Wales, Australia

^2^ Faculty of Medicine and Health, Specialty of Child and Adolescent Health, The University of Sydney

^3^ Marninwarntikura Women’s Resource Centre, Marulu Team, Fitzroy Crossing, Western Australia, Australia

^4^ Kid’s Research, Sydney Children's Hospitals Network, Westmead, New South Wales, Australia

^5^ Dalla Lana School of Public Health, The University of Toronto, Toronto, Ontario, Canada

*Corresponding Author

Anita Pickard, [anita.pickard@sydney.edu.au](mailto:anita.pickard@sydney.edu.au) , [0009-0006-5988-0120](file:///C:\Users\anitapickard\Library\Containers\com.apple.mail\Data\Library\Mail%20Downloads\408B951C-AA21-4B77-A96E-1106B1FA65B7\0009-0006-5988-0120)

Research projects led by MWRC and NCHS* in the Fitzroy valley were assessed using the Aboriginal and Torres Strait Islander Quality Appraisal Tool. Further discussion of the appraisal is included in Table 5, including an indication of how many ‘quality examples’ were met for each question. These examples are provided in the Aboriginal and Torres Strait Islander Quality Appraisal Tool companion document as guidance for interpreting and assessing research articles using the tool. Although not included as specific criteria, we report adherence to these examples here as a method for structuring the discussion.

**Table 5. Extension of Table 3. Aboriginal and Torres Strait Islander Quality Appraisal Tool**

| **Question** | **Lililwan*** | **Picture Talks*** | **Health Services** | | **Jandu Yani U** | | **Bigiswun Kid** | | **Marurra-U?** | |
| --- | --- | --- | --- | --- | --- | --- | --- | --- | --- | --- |
| Did the research respond to a need or priority determined by the community? | Yes (4/4). The Lililwan project was initiated by community leaders and researchers were invited to join in partnership, as leaders in Fitzroy Valley identified concerns about alcohol, FASD, ELT, and child health services. After the initial partnership between MWRC, NCHS*, The George Institute and the USYD formed in 2008, MWRC has continued to identify emerging priorities in child and family health including adolescent health and wellbeing, NDIS access, and trauma-informed care, and invited the USYD to partner on each subsequent research project. | | | | | | | | |  |
| Was community consultation and engagement appropriately inclusive? | Yes (3/3). Community engagement for each project was comprehensive, led by Aboriginal leaders, guided by community navigators and built on the relationship between MWRC, NCHS* and the USYD. Community navigators from each language group were included. This was possible due to Aboriginal leaders facilitating extensive community involvement beyond the ACCOs, with large teams of community members involved, consulting, and working on the research projects. Methods were designed in partnership with community leaders and navigators, including review of questions, translation and conduction of interviews. | | | | | | | | |  |
| Did the research have Aboriginal and Torres Strait Islander research leadership? | Yes (3/3). For each project, community leaders were included as chief investigators and co-researchers and named leads on ethics and grant applications. Aboriginal leaders and community members were also included as authors on all papers and project outputs. MWRC and NCHS* worked as the lead or joint lead partner organisations. Research implementation was led by MWRC and NCHS* and leaders had authority to make decisions and direct project outputs. | | | | | | | | |  |
| Did the research have Aboriginal and Torres Strait Islander governance? | Yes (2/2). MWRC and NCHS* guided planning of research and led the research implementation. The boards of these organisations oversaw the research, were consulted in the design, interpretation and dissemination and updated throughout the projects. Decision making was shared between organisations, with shared control and authority over the research process. Protocols were co-designed with MWRC and NCHS*, and ACCOs led research the implementation and process. Community consent (through ACCOs) was required for dissemination and publication of data and resources generated from research. | | | | | | | | |  |
| Were local community protocols respected and followed? | Yes (4/4). Aboriginal community navigators were employed to ensure all interactions with communities and participants were appropriate, and to cater for local language preferences. Data collection was guided by community navigators and remained flexible to accommodate for changes required for personal preferences of participants, or cultural reasons such as sorry business. Project materials such as the training program in Jandu Yani U were adapted for the local context. | | | | | | | | |  |
| Did the researchers negotiate agreements regarding rights of access to Aboriginal and Torres Strait Islander peoples’ existing intellectual and cultural property? | Partial (0/1). MoUs between project partner organisations outline roles and responsibilities for each organisation, outcomes and benefits of the research, respect of the culture and traditions of the Community, IP and data ownership and the principles of the collaboration. The MoUs specifically recognise Aboriginal and Torres Strait Islander Peoples’ inalienable right of ownership over their own existing lands, culture, knowledge and information and the value of collecting and recording Aboriginal health information is fully realised only when that information is owned by Aboriginal and Torres Strait Islander Peoples and their representative bodies and used to benefit them. However, the MoU is not legally binding, as preferred in the QAT. | | | | | Yes (1/1). Multi-institutional agreements between project partner organisations outline roles and responsibilities for each organisation, outcomes and benefits of the research, respect of the culture and traditions of the Community, IP and data ownership and the principles of the collaboration. Agreements specifically state the the ownership of Existing Material and Indigenous Cultural Heritage is not affected by the Agreement. | | | |  |
| Did the researchers negotiate agreements regarding rights of access to Aboriginal and Torres Strait Islander peoples’ ownership of intellectual and cultural property created through the research? | Partial (1/2). MoUs outline that data and IP created through the project will be owned by the partnership. MOUs outline that the Partners recognise and will work within the spirit of Aboriginal self-determination and community control. Published documents and studies do not specifically state the IP generated from the research is owned by Aboriginal and Torres Strait Islander peoples involved in the research. | | | | | Yes (2/2). Any data or IP developed from the project is owned by MWRC on behalf of the Fitzroy Valley community as outlined in the participant information sheets. | | Yes (2/2). Data and IP is owned by MWRC on behalf of the community as outlined in draft protocol paper and ‘ways of working’ document. | |  |
| Did Aboriginal and Torres Strait Islander peoples and communities have control over the collection and management of research materials? | Yes (3/3). Protocols were co-designed with MWRC and NCHS*. Collection was guided by community navigators, as described above. The community, through MWRC, had control of the research process. Approval for data usage and publication required agreement between MWRC and the USYD. Consent for use of the data was gathered in culturally appropriate ways, directed by community navigators, and gave participants control over data usage. Consent was project specific. | | | | | | | | |  |
| Was the research guided by an Indigenous research paradigm? | Partial (3/4). Extensive time was taken prior to the research to consult with community leaders and community members regarding community priorities and possible research implementation. The consultation was continued during implementation and informed research processes. Collaboration with community navigators embedded Aboriginal perspectives into planning, conduct, interpretation and dissemination of research and ensured engagement processes were appropriate. Through collaboration with Aboriginal leaders wholistic and complex concepts of health and wellbeing were acknowledged in project implementation, and guided identification of ongoing advocacy and research work. Although a Western research method was used, this was deemed necessary for the diagnostic prevalence study and recognised by all partners. | Yes (3/3). This study centered around Aboriginal ways of doing and knowing in regard to research. Conceptualisation of the study and development of research materials was codesigned by all parties, including Aboriginal leaders and community navigators. Yarning was used as the primary methodology and representation and dissemination of results involved creation of visual artworks. | | Partial (3/4). Conceptualisation of the study and development of research materials was conducted by all parties, including Aboriginal leaders and community navigators. Collaboration with community navigators embedded Aboriginal perspectives into planning, conduct, interpretation and dissemination of research and ensured engagement processes were appropriate. However, a specific Indigenous paradigm was not followed. | Yes (3/3). The Model of Engaging with Communities Collaboratively (MECC) was embedded in this project. Conceptualisation of the study and development of research materials was conducted by all parties, including Aboriginal leaders and community navigators. | | Yes (3/3). The Bigiswun Kid Project followed Aboriginal Participatory Action Research methodologies and principles. Ways of working documents for the Bigiswun Kid project explicitly emphasises the prioritization of Aboriginal ways of knowing and doing throughout the project. | | Yes (3/3). Research follows Aboriginal Participatory Action Research methodologies and principles. Conceptualisation of the study and development of research materials was conducted by all parties, including Aboriginal leaders and community navigators. The research also follows a grounded approach, practices are emergent and determined by MWRC and are flexible with Community priorities, responsive to the context. Multiple research methods used to capture complexity of health-wellbeing outcomes. | |
| Does the research take a strengths-based approach, acknowledging and moving beyond practices that have harmed Aboriginal and Torres Strait Islander peoples in the past? | Partial (2/3). | Yes (3/3) | | Partial (2/3) | Yes (3/3) | | | | | |
|  | A key strength of this research partnership is the strong relationship between organisations, and the prioritisation of building on this and working collaboratively. The partnership recognises harmful research practices of the past and the ongoing effects and trauma from colonisation. Each new project designed in accordance with ethical guidelines and reflecting the changes in Indigenous paradigms over time. The Lililwan project aimed to build upon the resilience of Fitzroy Valley community leaders in identifying and addressing FASD and early life trauma as priorities for their communities. Projects built the capacity of local organisations to act as service hubs and advocates for community health and wellbeing. However, results from the Lililwan and health services projects are deficits-focused as identifying challenges and needs was deemed necessary by the whole partnership team. Results from the Picture talks, Bigiswun Kid, Jandu Yani U and Marurra-U projects highlighted strengths and focused on solutions for communities. | | | | | | | | | |
| Did the researchers plan and translate the findings into sustainable changes in policy and/or practice? | Yes (3/3). Ongoing knowledge translation is described in table 2. | | | | | | | NA. Program implementation and research is underway. | |  |
| Did the research benefit the participants and Aboriginal and Torres Strait Islander communities? | Yes (4/4). Benefit to communities is described in Table 2. | | | | | | | Yes (4/4). Program implementation has begun with children receiving telecare, capacity building of education and health providers, and families attending therapeutic camps. | |  |
| Did the research demonstrate capacity strengthening for Aboriginal and Torres Strait Islander communities? | Yes (4/4). Community navigators were employed on all projects and local organisations employing Aboriginal people led each project. Local organisations and communities participated in activities to raise knowledge and awareness about FASD and early life trauma. Community navigators were employed and trained in research methodologies. | | | Yes (4/4). For each project the employment of community navigators facilitated two-way learning with navigators gaining training in research activities. Two full time staff in the Bigiswun Kid project were offered formal research training through TAFE. In the Jandu Yani U project, 38 local community members were trained and accredited by Triple-P International as parent coaches. MWRC staff undertaking university studies (including in Social Work) are supported through implementing partner organisations such as RFW. Throughout the partnership, MWRC has remained and grown as a lead support organisation for the community. | | | | | |  |
| Did everyone involved in the research have opportunities to learn from each other? | Yes (4/4). All non-Aboriginal members of research teams engaged with cultural safety training. Aboriginal leaders guided new members by sharing local histories and cultures prior to project work. Aboriginal staff received training in research activities and there was an ongoing sharing of project results and findings. Researchers in partnership with local organisations presented findings back to communities and participants. The Marurra-U ways of working document highlights two-way learning as a key principle of the partnership. | | | | | | | | |  |
